# On the reliability of value-modulated attentional capture: An online replication and multiverse analysis

**DOI:** 10.3758/s13428-023-02329-5

**Published:** 2024-01-09

**Authors:** Francisco Garre-Frutos, Miguel A. Vadillo, Felisa González, Juan Lupiáñez

**Affiliations:** 1 Mind, Brain and Behavior Research Center (CIMCYC), University of Granada, Campus of Cartuja, 18011 Granada, Spain; 2 Department of Experimental Psychology, University of Granada, Granada, Spain; 3 Department of Basic Psychology, Faculty of Psychology, Autonomous University of Madrid, Madrid, Spain

**Keywords:** Value-modulated attentional capture, Visual search, Learning, Multiverse, Reliability

## Abstract

Stimuli predicting rewards are more likely to capture attention, even when they are not relevant to our current goals. Individual differences in value-modulated attentional capture (VMAC) have been associated with various psychopathological conditions in the scientific literature. However, the claim that this attentional bias can predict individual differences requires further exploration of the psychometric properties of the most common experimental paradigms. The current study replicated the VMAC effect in a large online sample (*N* = 182) and investigated the internal consistency, with a design that allowed us to measure the effect during learning (rewarded phase) and after acquisition, once feedback was omitted (unrewarded phase). Through the rewarded phase there was gradual increase of the VMAC effect, which did not decline significantly throughout the unrewarded phase. Furthermore, we conducted a reliability multiverse analysis for 288 different data preprocessing specifications across both phases. Specifications including more blocks in the analysis led to better reliability estimates in both phases, while specifications that removed more outliers also improved reliability, suggesting that specifications with more, but less noisy, trials led to better reliability estimates. Nevertheless, in most instances, especially those considering fewer blocks of trials, reliability estimates fell below the minimum recommended thresholds for research on individual differences. Given the present results, we encourage researchers working on VMAC to take into account reliability when designing studies aimed at capturing individual differences and provide recommendations to improve methodological practices.

**Supplementary Information:**

The online version contains supplementary material available at 10.3758/s13428-023-02329-5.

## Introduction

Classical theories of attentional orienting often describe two primary sources of attentional control. Attention can be directed by our goals, intentions, or specific task demands (Folk et al., 1994; Folk & Remington, 1998), or by the physical features of stimuli (Theeuwes, 1992, 1994). However, not all effects related to attentional orienting fit well with this classification and it has been suggested that the distinction between goal-directed and stimulus-driven attentional control^1^ is in fact a 'failed theoretical dichotomy' (Awh et al., 2012). There is compelling evidence that our learning history with stimuli can modulate attentional priority in ways that are neither clearly goal-directed or stimulus-driven. This constitutes a third source of attentional control, which is often referred to as “selection history”. The concept of selection history comprises a set of phenomena in which attention is biased towards stimuli with which we have previous experience, which are no longer relevant to our goals, but are not necessarily salient either, so they cannot be framed in the traditional theoretical dichotomy outlined above (Anderson et al., 2021; Awh et al., 2012; Theeuwes, 2018). Selection history is a broad overarching construct that includes different influences over attentional control based on implicit learning mechanisms. Examples of selection history include, for instance, our ability to search for a target (or ignore a distractor) exploiting regularities across trials (Chun & Jiang, 1998; Geng & Behrmann, 2002; Wang & Theeuwes, 2018), the fact that it is easier to search for a stimulus when has been selected for response in preceding trials (Found & Müller, 1996; Maljkovic & Nakayama, 1994, 1996), or automatic attentional biases towards arbitrary features of a stimulus that are associated with the prospect of reward (Anderson et al., 2011b; Della Libera & Chelazzi, 2009; Le Pelley et al., 2015).

The latter instance of selection history is known as reward history. One of the earliest demonstrations of this attentional bias was reported by Anderson et al. (2011a). For their study, they designed an experimental paradigm based on the Additional Singleton task (Theeuwes, 1992), where participants were first presented with a visual display and had to find one of two colors, which were associated with different reward magnitudes during a training phase. During a subsequent test phase, participants were asked to find targets based on their shape, therefore rendering color irrelevant. Crucially, the colors that had been targets in the training phase were now presented as distractors. Their results showed that there was an increment in response times (RTs) when the high value stimulus acted as a distractor, compared to when the low valued stimulus was present. This effect was later termed value-modulated attentional capture (VMAC).

These results show that irrelevant features of stimuli that have been associated with rewards are more likely to capture attention, even when they are no longer predictive of rewards. Although this effect could not be attributed to physical salience or task goals, the study by Anderson et al. (2011a) did not prove that the VMAC effect is independent of previous task relevance, because color, while not task-relevant in the test phase, was relevant during the training phase. In other words, the effect could be driven by an automated instrumental response to the features that were relevant during the training stage, regardless of their associations with reward. To demonstrate that VMAC is indeed independent of task relevance, Le Pelley et al. (2015) introduced a slight modification to Anderson et al.'s (2011a) paradigm. Specifically, they removed the training phase where color is the target defining feature and introduced reward feedback that depended on distractor color in the test stage. In other words, the stimulus predicting the reward always played the role of a singleton color distractor that participants had to ignore to search for the shape defined target, from the beginning of the experiment. With this alternative procedure, Le Pelley et al. (2015) showed that VMAC could still emerge in conditions where color is an irrelevant feature during the whole task. Furthermore, Le Pelley et al. (2015) found that the high-value distractor still captured attention even under conditions where fixating the eyes on it actually prevented the delivery of the reward. This led to participants earning fewer rewards than they potentially could have.

Le Pelley et al. (2015) showed that the acquisition of the VMAC effect cannot be attributed to previous task relevance. However, while in Le Pelley et al. (2015) color was task irrelevant in the sense that color acted as a distractor, color did nevertheless provide participants with useful information about how much reward they could expect to obtain from each trial. This opens the possibility that the observed attentional capture effects derive from an explicit strategy rather than automatic attentional capture by high-reward predictive stimuli. To establish that the VMAC effect is not dependent on informational value, Watson et al. (2019a) combined the two paradigms previously mentioned. In their study, the paradigm of Le Pelley et al. (2015) served as an initial training phase, to observe how the VMAC effect emerged and developed throughout the task. In a later phase, participants were explicitly informed that color would not be associated with reward anymore, and then continued the task without reward feedback, like the test phase in the Anderson et al. (2011a) paradigm. This adaptation by Watson et al. (2019a) showed that the VMAC effect persisted even when color was no longer predictive of reward. In other words, Watson's version of the task confirms that VMAC can be observed even when paying attention to reward-related stimuli has never been instrumental for successful performance in the task and these stimuli no longer provide useful information about the size of the reward. These two features render Watson's paradigm ideal to isolate the core components of VMAC.

Since Anderson et al.’s (2011a) seminal work, this type of attentional bias has been observed in both overt and covert attention measures (Anderson, 2015; Bucker et al., 2015; Le Pelley et al., 2015; Theeuwes & Belopolsky, 2012; Watson et al., 2020; Watson et al., 2019b); it seems to be robust to extinction (Anderson & Yantis, 2013) and resistant to cognitive control (e.g. explicit instructions to ignore distractors, Pearson et al., 2015). Given these characteristics, some researchers have linked the VMAC effect with a form of human sign-tracking, or a tendency to endow a Pavlovian signal of reward with incentive salience (Berridge et al., 2009), eliciting automatic attentional approach responses to the associated feature (Flagel & Robinson, 2017; Robinson & Flagel, 2009). Given that individual differences in propensity to sign-tracking behavior are theoretically related to behavioral disorders (Colaizzi et al., 2020; Flagel et al., 2009) and that attentional biases are believed to play a critical role in psychopathological conditions such as substance abuse (Field & Cox, 2008), there have been numerous attempts in the literature to establish a link between the VMAC effect and different psychopathological conditions (Anderson, 2021). For instance, individual differences in various aspects of VMAC effect have been linked to depressive (Anderson et al., 2014, 2017) and obsessive-compulsive symptoms (Albertella et al., 2020a, 2020b; Basel & Lazarov, 2022) in non-clinical samples, attention deficit hyperactivity disorder (ADHD) symptoms in children (Sali et al., 2018), substance abuse (Albertella et al., 2017, 2019, 2021; Anderson et al., 2013; Liu et al., 2021), risk-taking behavior in individuals with HIV (Anderson et al., 2016) and individual differences in working memory capacity and cognitive control (Anderson et al., 2011b, 2013, 2016; Anderson & Yantis, 2012).

Given how often different studies have associated this effect with diverse psychopathological conditions, we would be tempted to consider the VMAC effect as a valid measure of sign-tracking. However, a measure cannot be valid if it is not reliable (Loevinger, 1957). Traditionally, the use of cognitive-behavioral measures has tended to overlook issues of measurement error, thus neglecting the impact of reliability on inferences drawn from individual differences studies (Hedge et al., 2018). In fact, when reported, the reliability of cognitive measures of attentional bias to reward predictive stimuli tends to be low (Ataya et al., 2012), a fact that has significant consequences on potential inferences from these measures. These consequences range from attenuated correlations with other measures to unpredictable effects on statistical power depending on the ratio between true variance and error variance (De Schryver et al., 2016; Zimmerman & Zumbo, 2015) or incomparable effect sizes between populations or even different studies (Cooper et al., 2017).

To the best of our knowledge, only a handful of studies have investigated the reliability of the VMAC effect. Anderson and Kim (2019) assessed the test–retest reliability of the attentional capture in Anderson's paradigm. Their participants underwent a training phase to learn color reward contingencies, followed by a test phase immediately after training and a delayed test 1 week later. While the test reliability of the effect was quite good when measured with eye movements (*r* = .80), reliability was disappointingly low when using RTs as dependent measure (*r* = .12). With regards to the Le Pelley’s paradigm, a recent study by Freichel et al. (2023) reported that the test–retest reliability, measured with RTs, was again quite low (*r* = .09). These two studies suggest that measures based on RTs may not be suitable for studying individual differences (see Draheim et al., 2019 for a discussion). Most importantly, these two studies have explored the test–retest reliability of VMAC, that is, the temporal stability of the effect. However, some researchers argue that defining reliability as temporal stability is not particularly informative in learning paradigms, where measures are expected to change over time. In such contexts, internal consistency, or the degree to which different parts of the same test measure the same thing, may be more informative (Farkas et al., 2023). Unfortunately, the internal consistency of the VMAC effect has not yet been assessed in either of the two paradigms mentioned above.

In light of the above, the present study has two main aims. First, we sought to replicate and extend the results of Watson et al. (2019a). As explained above, Watson et al. (2019a) combined the two paradigms used by previous studies. In this variant of the task, the VMAC effect can be measured while learning is still in progress (i.e., the rewarded phase, equivalent to the learning phase in Le Pelley’s paradigm), and also once the associated feature no longer holds informational value (i.e., the unrewarded phase, equivalent to the test phase in Anderson’s paradigm). In the original study by Watson et al. (2019a), the unrewarded phase was relatively brief compared to the rewarded phase (only two blocks of 24 trials compared to 12 blocks in the rewarded phase). Here, we increased the length of the unrewarded phase to match the length of the rewarded phase. This allowed us to explore whether the VMAC effect persists once reward feedback is eliminated. The second aim of this study was to test the internal consistency of VMAC scores in both stages of Watson et al.'s procedure, which combines Anderson's and Le Pelley's paradigms. In addition, given that many correlational (Albertella et al., 2020a, 2020b, 2021; Liu et al., 2021) and experimental studies (Le Pelley et al., 2022; Watson et al., 2020) have been conducted online, which is especially useful when large samples are needed, we decided to run an online version of the task. Finally, sometimes researchers must face the plethora of decisions of possible specifications over data preprocessing, the so-called *garden of forking paths* (Gelman & Loken, 2013). Different combinations of data processing pipelines can yield radically different results, with dramatic implications on statistical inferences and also on the reliability of the measures employed. To assess the impact of these arbitrary preprocessing decisions, we calculated reliability estimates for different combinations of data preprocessing specifications (i.e., specification curve or multiverse analysis; Simonsohn et al. 2020; Steegen et al., 2016).

## Method

### Participants

Potential participants were contacted through the distribution lists of the University of Granada. From the group of undergraduate students who showed an interest in participating, 216 participants actually conducted the experiment. All of them had normal or corrected-to-normal vision and were naive as to the purpose of the experiment. Participants were informed that based on performance they could earn up to €10. The study was approved by the Ethical Review Committee of the University of Granada.

Of the 216 participants who chose to participate in the study, 23 did not complete the whole experiment and were removed from the analysis. Of the remaining 193 participants, we filtered out those with an accuracy lower than 70%. The final sample consisted of 182 participants (60 males; *M*_age_ = 21.9; *SD*_age_ = 3.3).

#### Stimuli, design, and procedure

Considering that the eccentricity of items in the search display is an important factor in this paradigm, to adjust the dimensions of the stimuli to participants’ conditions, we estimated the distance at which each participant was located from the screen using the virtual chinrest procedure developed by Li et al. (2020). Before starting the experiment, participants were instructed to fit an object with a standard size (i.e., a credit card or a driver’s license) to a rectangle on the computer screen, whose size they could change using two buttons from the keyboard. Second, to estimate the location of their visual blind spot, participants were asked to cover their right eye while looking with their left eye at a fixed placeholder that appeared in the center of the monitor. Meanwhile, a red circle moved to the left and participants were instructed to press the spacebar when they noticed that the circle disappeared. Blind spot estimation was based on five repetitions of this procedure, where the average of these five repetitions was employed to calculate screen distance.

**Fig. 1:**
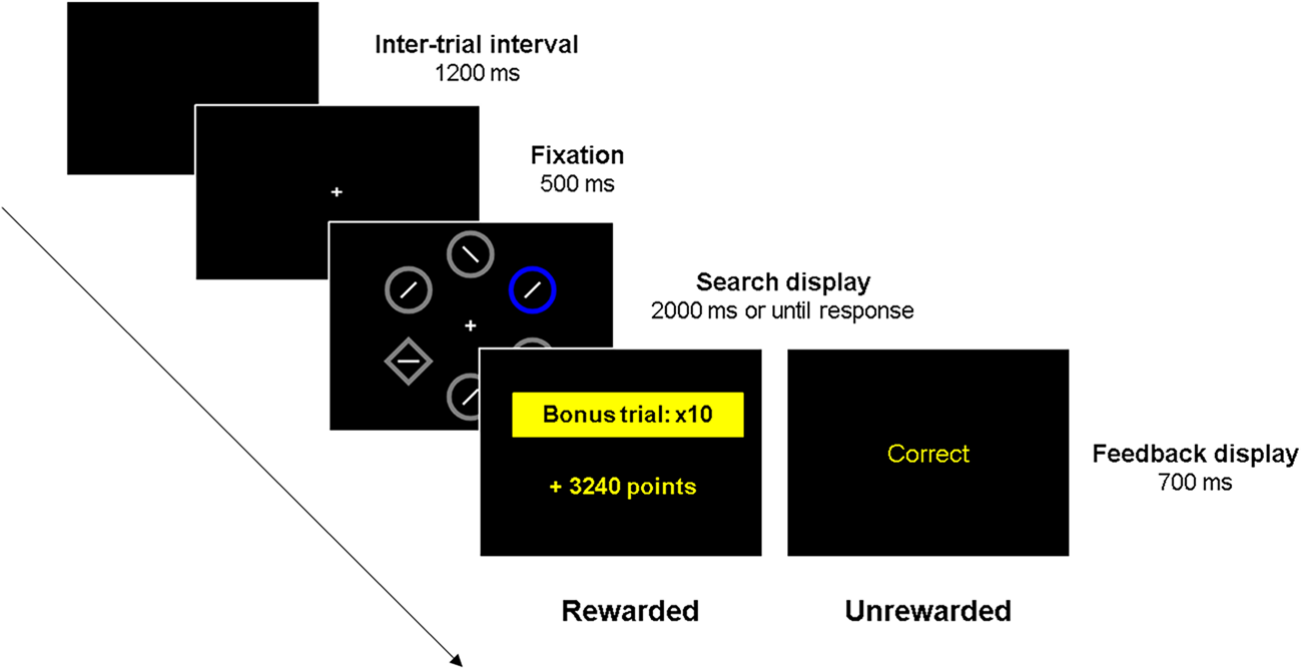
Graphical representation of the experimental procedure. Example of the sequence of events in the experimental task. In the first phase of the experiment (rewarded phase), participants could earn points based on performance, and when a high reward singleton appeared in the display, points were multiplied by 10. In the second phase (unrewarded phase), participants were neither rewarded nor punished based on either performance or singleton color, only accuracy feedback was provided. Feedback was provided in Spanish

The experimental task was adapted from previous studies reported in Le Pelley et al. (2015) and Watson et al. (2019a), was programed in OpenSesame (Mathôt et al., 2012) and hosted in JATOS (Lange et al., 2015). A graphical representation of the procedure is presented in Fig. 1. Each trial started with a central fixation cross, followed by a search display containing six shapes (2.3° × 2.3° visual angle) evenly arranged around an imaginary circle (10.1°). Five of the shapes were circles, each containing a segment tilted 45° randomly to the left or right. The target was a diamond containing a segment oriented randomly horizontally or vertically. In most trials, one of the circles was colored, while the other shapes were grey. For some participants, the colored circles were blue and orange, and for others, they were green and pink. The colors of the high- and low-reward circles were randomly assigned. The location of the target and the distractor were random on each trial.

Participants were instructed to indicate, as quickly as possible, the orientation of the segment inside the diamond, by pressing either ‘V’ for horizontal or ‘H’ for vertical, with faster responses earning more points. Each block included 24 trials, comprising ten trials with a distractor in the high-reward color (high singleton condition, ten trials with a distractor in the low-reward color (low reward condition), and four distractor-absent trials (absent singleton condition) where all shapes were grey. During the first part of the task (rewarded phase) participants were awarded 0.1 points for every millisecond that their RTs was below 1000 ms on low-reward-distractor trials. On high-reward trials, the points were multiplied by 10. Responses with RT greater than 1000 ms were awarded no points, and errors led to the loss of the same number of points that would have been earned. The search display remained on screen until the participant responded, or the trial timed out after 2000 ms. Feedback was then provided for 700 ms, indicating the number of points won or lost for correct and incorrect responses, respectively. The inter-trial interval was 1200 ms. In the last phase of the task (unrewarded phase), participants could not earn points based on performance, and only accuracy feedback was presented.

After the calibration described above, participants completed a small practice phase of 24 trials. In this phase, in 20 trials a singleton appeared in a different color from the one used in the main experimental task, while in the rest of the trials the singleton distractor was absent. Afterwards, instructions informed participants that in the following phase of the experiment (i.e., the rewarded phase) they could earn points based on their performance. Participants were instructed that faster and correct responses would result in more points, and when the high-colored singleton appeared on the screen there would be a “bonus trial”. Then, participants completed the rewarded phase with 12 blocks of 24 trials (288 trials). Once the rewarded phase finished, participants were instructed that no more points would be available in the following, unrewarded phase and that they should continue responding to the orientation of the line within the diamond as quickly and accurately as possible. Participants then completed 12 blocks of 24 trials for a total of 288 trials in the unrewarded phase.

## Results

### Experimental analysis

To estimate how VMAC changed over blocks and across phases, we used linear mixed models (LMMs). LMMs offer several advantages, such as avoiding the need for data aggregation and handling unbalanced data effectively. These models are particularly well suited for analyzing longitudinal data, providing flexibility in capturing nonlinear relationships by allowing transformations of both the response and predictors.

As suggested by Barr et al. (2013), we fitted the maximal random effect structure that does not result in convergence issues (Bates et al., 2015; Matuschek et al., 2017). We refer to this model as the maximal feasible model. We fitted different LMMs for each phase starting from the following maximal model structure^2^:

$$\mathit{\log}(RT)\sim Singleton\ast Block+\left( Singleton\ast Block\ |\ Participant\right)$$

where RTs are log-transformed to approximate normality. For both stages, we set the hypothesis matrix for the Singleton predictor to have coefficients for high-low singleton and for low-absent singleton through repeated contrasts, thus allowing us to explore both the VMAC effect (high-low) and the attentional capture effect (low-absent). Furthermore, we centered the block predictor to facilitate interpretability. For models regarding each phase, we discarded the first two trials of each block and we excluded incorrect responses (rewarded phase: 5.99%; unrewarded phase: 5.50%) and RTs below 150 ms or RTs above 1800 ms ( < 1% in both phases).

**Fig. 2:**
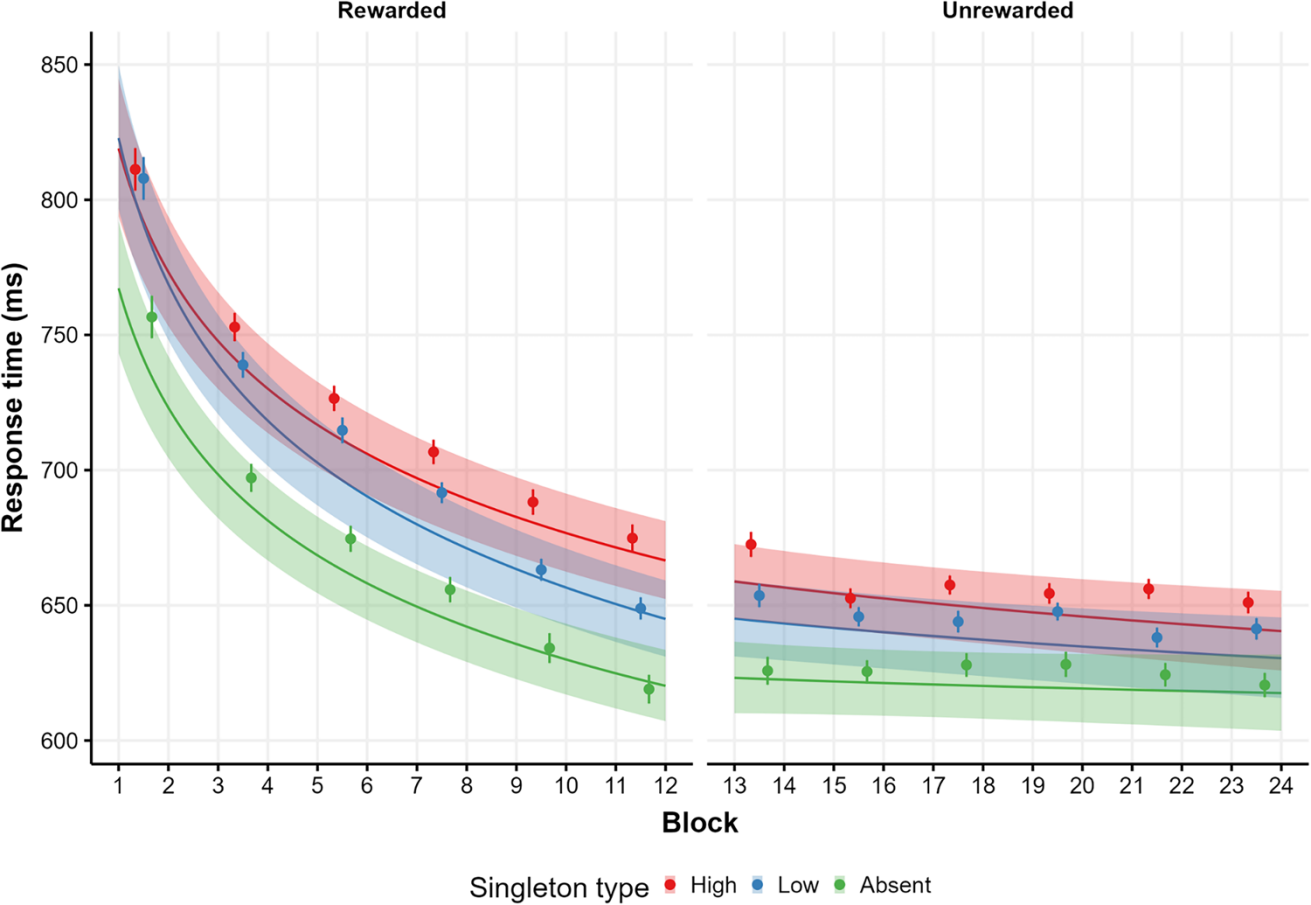
Model predictions for each type of singleton across blocks and phases. This figure shows model predictions across types of singleton, blocks, and phases, where each phase is modeled independently. *Lines* show the predicted conditional means for each singleton, while *shaded areas* denote 95% CI. *Dots* indicate the observed means across epochs of two blocks, and *error bars* represent the standard error of the mean (SEM)

**Fig. 3:**
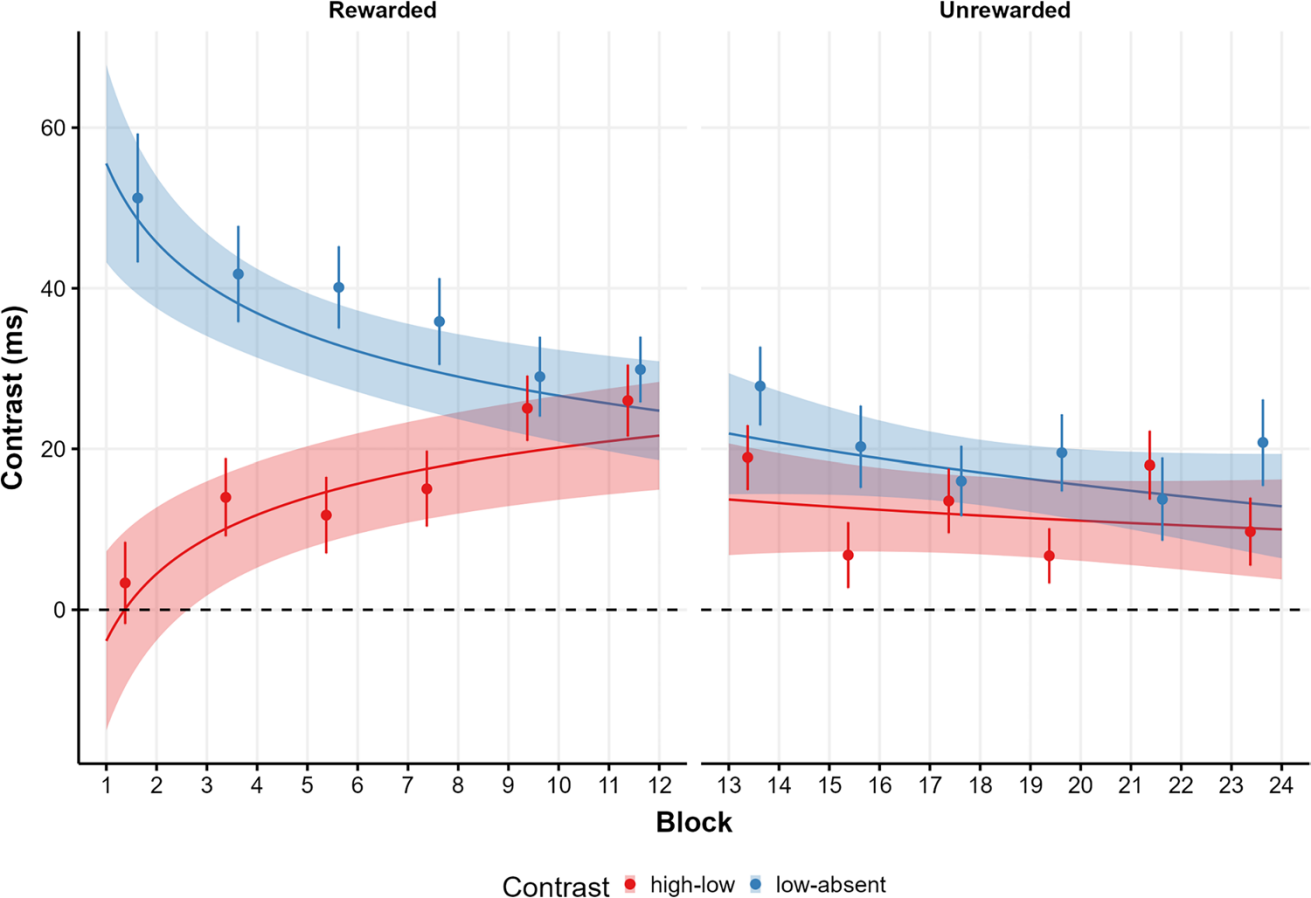
Conditional effect of high-low and low-absent through blocks. This figure illustrates the conditional effects of singleton across blocks and phases, where each phase represents different models. *Colors* distinguish predictions for two contrasts: the high-low (VMAC effect) and the low-absent (attentional capture effect). *Lines* show the predicted conditional means for each effect, while the *shaded areas* denote 95% CI. *Dots* indicate sample means of epochs of two blocks, and *error bars* represent SEM

In the *rewarded phase***,** given that the maximal model failed to converge, we dropped the Singleton*Block interaction from the random effect structure. We compared the linear version of the maximal feasible model with a power function (achieved by transforming the block predictor to logarithm; Wang et al., 2020). We chose the power function model due to its lower *AIC* ( ∣*Δ*_*AIC*_ ∣  = 517.6). Model coefficients are presented in Table 1 and model predictions^3^ are presented in Fig. 2 (left side). As can be seen, the predictor for the high-low contrast was significant, with higher RTs for high singleton trials (*M* = 713, 95% CI [698, 729]) than low singleton trials (*M* = 699, 95% CI [683, 715]), showing a VMAC effect (*M*_high-low_ = 14 , 95% CI [8, 20]). The predictor for low-absent singleton was also significant, as low singleton trials show higher RTs than absent trials (*M* = 665, 95% CI [651, 679]), reflecting attentional capture when the low color singleton was presented (*M*_low-absent_ = 34, 95% CI [29, 39]). Moreover, the block predictor was significant, meaning that RTs reduced over blocks. Of more interest are the interactions between block and singleton contrasts, showing that while the low-absent singleton contrast decreased over blocks, the high-low contrast increased. Figure 3 shows the conditional effect of high-low and low-absent through the blocks of the rewarded phase. As can be seen, model predictions indicate that the VMAC effect starts to be significant after three blocks of trials.

**Table 1:** Model summaries for the selected models in both the rewarded and unrewarded phase

	Rewarded phase			Unrewarded phase		
Predictors	Estimates	CI	*p*	Estimates	CI	*p*
(Intercept)	6.516	6.495–6.537	**< .001**	6.431	6.411–6.451	**< .001**
High-low	.021	.012–.030	**< .001**	.018	.011–.026	**< .001**
Low-absent	.049	.042– .056	**< .001**	.027	.021–.033	**< .001**
Block	– .063	– .071 to – .056	**< .001**	– .006	– .011 to – .002	**.002**
High-low × Block	.011	.007–.015	**< .001**	– .002	– .006 to .003	.449
Low-absent × Block	– .009	– .015 to – .003	**.002**	– .004	– .010 to .001	.134
Random effects						
σ^2^	.046			.045		
τ_00_	.021			.019		
τ_11_	.003_ high-low_			.002 _high-low_		
	.001_ low-absent_			.000_ low-absent_		
	.002 _Block_			.001_ Block_		
ρ_01_	– . 102			– . 173		
	.369			.734		
	– . 265			.220		
ICC	.350			.314		
N	182			182		
Observations	45089			45309		
Marginal *R*^2^ / Conditional *R*^2^	.063 / .391			.005 / .317		

Bold entries shows statistical significance

*p*-values were computed using Satterwhite correction. CI = confidence interval; ICC = intraclass correlation coefficient. σ^2^ = model residuals, τ = random effects, ρ = correlation between random effects

The same analysis was carried out for the *unrewarded phase*. The maximal feasible model was, again, the model including only the random slopes of singleton and block. Then, we compared the linear model with the power function model, where the latter was selected due to its lower AIC (∣*Δ*_*AIC*_ ∣  = 23). Model coefficients are presented in Table 1 and model predictions are presented in Fig. 2 (right side). As can be observed, the predictor for the high-low contrast was significant, with longer RTs for high singleton trials (*M* = 649, 95% CI [636, 662]) than low singleton trials (*M* = 637, 95% CI [624, 651]), resulting in a VMAC effect (*M*_high-low_ = 12, 95% CI [7, 17]). The predictor for low-absent was also significant, as low singleton trials exhibit higher RTs than absent trials (*M* = 620, 95% CI [608, 632]), again, indicating attentional capture when the color singleton is presented (*M*_low-absent_ = 17, 95% CI [13, 21]). Lastly, the block predictor was significant, with a progressive reduction of RTs across blocks. Finally, neither the high-low or the low-absent contrasts interacted with block suggesting that neither the VMAC effect nor the attentional capture effect changed throughout the entire unrewarded phase. A paired sample *t* test averaging data from the last two blocks confirmed that the VMAC effect was still present at the end of the unrewarded phase (*M*_high-low_ = 9.74, 95% CI [.64, 18.83], *t*(181) = 2.11, *p* = .036; *d* = .16, 95% CI [.01, .30]).

To test whether the effect actually changed between the rewarded and unrewarded phase, in the same vein to the original Watson et al. (2019a), we compared the VMAC effect of the averaged data of the last two blocks of the rewarded phase in comparison to the first two blocks of the unrewarded phase. To that aim we ran a repeated measure ANOVA with singleton (high, low) and phase (rewarded, unrewarded) over RTs. The results showed a significant effect of singleton (*F*(1, 181) = 39.74, *MSE* = 2312.27, *p* < .001, *η*_*p*_^*2*^ = .180), but no effect of phase (*F*(1, 181) = 0.10, *MSE* = 2813.98, *p* = .757, *η*_*p*_^*2*^ < .001) nor a singleton*phase interaction (*F*(1, 181) = 1.68, *MSE* = 1351.12, *p* = .196, *η*_*p*_^*2*^ = .09). These results support the conclusion that the omission of reward and explicit instructions does not significantly modulate the VMAC effect, and directly replicate Watson et al. (2019a) main finding.

Finally, to test whether the previous results could have been driven by a speed–accuracy trade-off, we fitted two models with the same structure as those for the rewarded and unrewarded phases to analyze accuracy, using a binomial distribution with a logit link. To overcome convergence problems, we dropped the singleton predictor for the random structure from the rewarded phase model. As can be seen in Table 2, both models reveal a progressive increase in accuracy over time. In the rewarded phase (left side of Table 2) there is also a significant effect in the low-absent contrast, due to the fact that accuracy for low singleton trials (*Accuracy* = .955, 95% CI [.949, .960]) is lower than accuracy for absent trials (accuracy = .960, 95% CI [.954, .966]), but this difference is numerically small (𝛥*Accuracy*_low-absent_ **= –** . 005, 95% CI [– . 010, – . 001]). In fact, based on Chen (2010), the effect size (odds ratio; *OR*) for this contrast is very small (1/*OR* = 1.16, 95% CI [1.03, 1.30]). Critically, none of the model coefficients regarding high-low nor its interaction with block were significant, which suggests that the previous results are not contaminated by speed–accuracy trade-offs.

**Table 2:** Model summaries for the accuracy analysis

	Rewarded phase			Unrewarded phase		
Predictors	Estimates	CI	*p*	Estimates	CI	*p*
(Intercept)	22.730	20.186–25.595	**< .001**	23.317	20.573 – 26.428	**< .001**
High-low	1.033	.948–1.127	.459	1.005	.893–1.131	.934
Low-absent	.864	.767–.974	**.016**	1.043	.890–1.222	.601
Block	1.221	1.142–1.306	**< .001**	1.066	1.009–1.127	**.022**
High-low × Block	.981	.907–1.061	.639	.985	.900–1.079	.748
Low-absent × Block	.958	.862–1.065	.430	.910	.809–1.023	.113
Random effects						
σ^2^	3.290			3.290		
τ_00_	.532			.592		
τ_11_				.087_ high-low_		
				.184_ low-absent_		
	.103_ Block_			.015_ Block_		
ρ_01_	– . 160			– . 099		
				– . 016		
				.071		
ICC	.162			.161		
N	182			182		
Observations	47807			47821		
Marginal *R*^2^ / Conditional *R*^2^	.010 / .170			.001 / .162		

Bold entries shows statistical significance

CI = confidence interval; ICC = intraclass correlation coefficient. σ^2^ = model residuals,  τ = random effects, ρ = correlation between random effects

### Multiverse reliability analysis

As explained above, we generated different datasets with specifications under a combination of various factors that could potentially affect the internal consistency of the effect of interest. First, sometimes participants with near chance accuracy are removed from the analysis (Albertella et al., [Bibr CR5][Bibr CR1], 2020b; Liu et al., 2021). However, given task performance is linked to the experimental manipulation, we wanted to explore if using different thresholds to select participants could have a significant impact on reliability. To explore this possibility, we excluded participants whose mean proportion of accuracy was lower than either .50 or .70.

Regarding task length, previous studies differ substantially in the number of trials included in the analysis. For example, in studies using the Anderson's paradigm, singleton absent trials have to be excluded, often resulting in a loss of 50% of trials (Anderson et al., 2013, 2014, 2016). In the same vein, in the Le Pelley’s paradigm it is common to use only a subset of the trials available, due to the fact that researchers are often interested in measuring the effect in late stages of learning, where the effect is expected to be larger (Albertella et al., 2019, 2020b; Liu et al., 2021), or because a subsequent test phase is used with a reduced number of trials (Albertella et al., 2019, 2020a). Typically, this strategy would lead to a task length of roughly two or three blocks of trials in the present study (40–60 trials). Given that the number of trials is usually positively related to reliability in behavioral–cognitive measures (Hedge et al., 2018; von Bastian et al., 2020), this practice might compromise the reliability of VMAC scores. To explore this possibility, we manipulated whether different numbers of blocks of each phase were included. For the rewarded phase, we used either the last two blocks of trials, the last half of the task (six blocks) or the whole phase. For the unrewarded phase, we selected either the first two blocks of trials, the first half of the phase or the whole phase.

Our multiverse analyses also considered different approaches to filtering RTs. This filter could be fixed (deciding to eliminate from the analyses RTs that could be considered too fast or too slow, compared to a fixed RT, which represents the construct of interest) or relative (eliminating trials where RTs are above or below each participant's and condition mean). In the literature, fixed or relative filters are often employed, sometimes arbitrarily, under the assumption that some extreme RTs may introduce noise in the analysis. In contrast, a recent study has shown that using any type of filter over RTs induces bias in the estimates, and severely reduces statistical power (Miller, 2023). However, although the use of procedures of outlier removal may be harmful for experimental research, it may have different consequences in correlational research. Here, to explore the impact of different approaches of outlier removal procedures, we orthogonally varied whether different combinations of either fixed or relative filters were applied. First, we manipulated whether a fixed filter (i.e., removing RTs below 150 ms or higher than 1800 ms) was applied or not. Secondly, we also manipulated whether a relative filter was applied or not and the severity of the filter. Specifically, we filtered trials two or three standard deviations away from each participant's mean or, alternatively, we did not apply any relative filter. In addition, in some studies the first two trials of each block are eliminated on the assumption that these trials produce more noise as participants are still not engaged in the task (Le Pelley et al., 2015; Watson et al., 2019a). To test whether this decision can help improve reliability estimates, we manipulated whether the first two trials of each block were filtered out or not.

Lastly, the most common approach to compute the VMAC effect is through a difference of means for the high and low singleton trials. Given that the distribution of RTs is positively skewed, the mean could be a biased estimate of the central tendency of the distribution. For that reason, a more robust statistic, such as the median, could be computed. On the other hand, it is possible to take the logarithm of the RTs to normalize the distribution. We compared these possible approaches, using either the raw RTs or log transformed RTs, and either the mean or the median as the averaging method of the RTs distribution.

To sum up, our multiverse analysis included the possible specifications by combinations of the following factors:

- Eliminate participants based on accuracy cut-off: < 50% or < 70%.
- Relative filter for RTs: none, 2 SDs or 3 SDs.
- Fixed filter for RTs: none or (RT > 150 and RT < 1800),
- Averaging method: mean or median.
- Log-transform RTs: yes or no.
- Filter the first two trials of each block: yes or no.
- Number of blocks used to calculate the effect: 2, 6, or 12.

The combination of all the possible levels of these factors results in 288 possible specific datasets. For each dataset, we calculated the reliability separately for each phase (rewarded and unrewarded). Furthermore, as the present paradigm also allows for the calculation of the attentional capture effect (low-absent contrast), we also report the results of a multiverse analysis of this effect in the Supplementary Material.

For the computation of reliability estimates, we used split-half correlations to estimate internal consistency. Instead of employing an arbitrary split-method (such as odd vs. even trials, or first vs. second half), we used a permuted random split procedure. In this procedure, all the trials were randomly split into two halves, always ensuring an equal number of trials of each block in each half, and a difference score (high-low) was computed for each half. Then, we calculated a Pearson's *r* correlation for the difference score in each half, and applied the Spearman–Brown correction formula (Spearman, 1910) to that correlation. This procedure was permuted 10,000 times, and the mean Spearman–Brown estimate of the distribution of permutations was taken as the reliability estimate, with the 2.5th and 97.5th quantiles as the 95% bootstrapped CI.

**Fig. 4:**
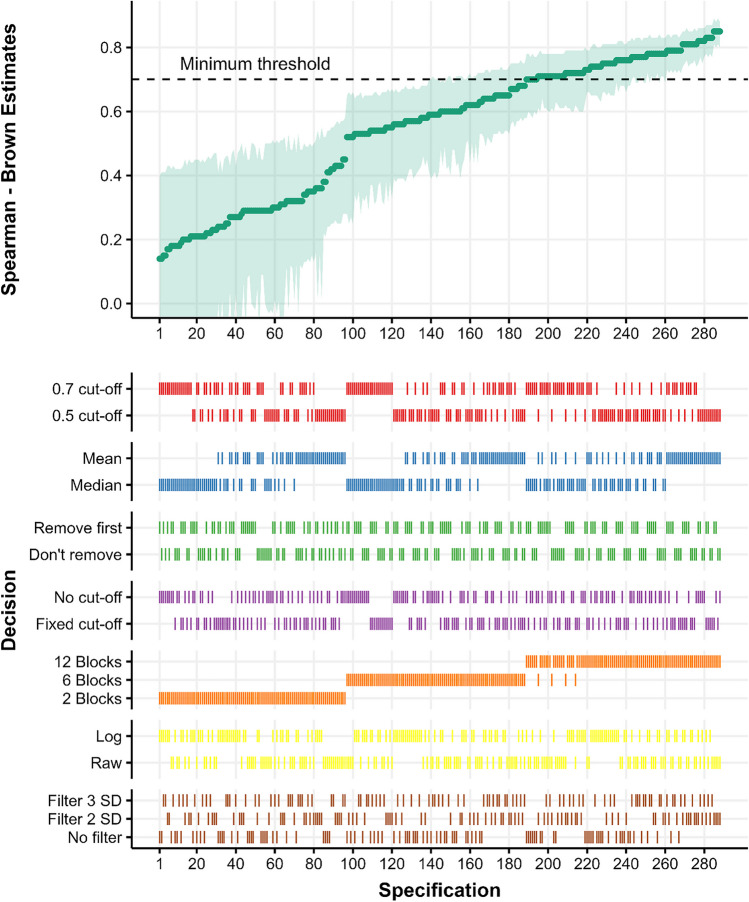
Spearman–Brown estimates across the rewarded phase. In the top panel, each *dot* represents a Spearman–Brown reliability estimates for the rewarded phase, and *shaded areas* represent 95% CI. In. In the bottom panel, the different combinations of specifications are signaled with a *vertical line*. The *shaded line* in the top panel highlights .7, as the minimum threshold for studies on individual differences

**Fig. 5:**
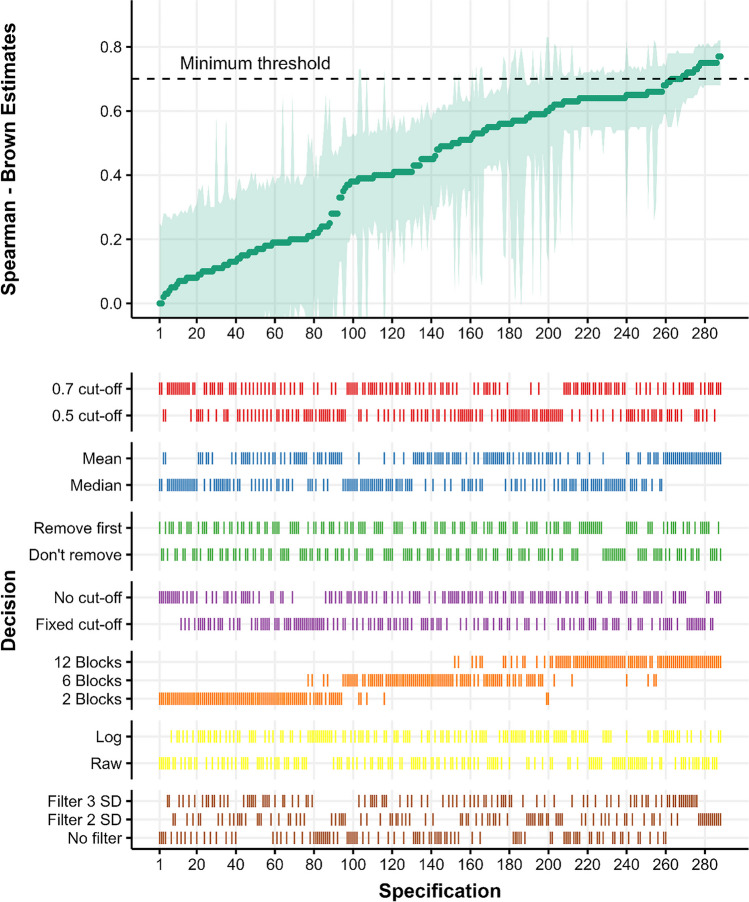
Spearman–Brown estimates across the unrewarded phase. In the top plot, each *dot* represents a Spearman–Brown reliability estimate for the unrewarded phases, and the *shaded areas* represent 95% CI. In. In the bottom plot, the different combinations of specifications are signaled with a *vertical line*. The *shaded line* in the bottom plot highlights .7, as the minimum threshold for studies on individual differences

The results of the multiverse analysis for the rewarded and unrewarded phases are shown in Figs. 4 and 5, respectively. Both figures show the curve of reliabilities sorted in ascending order (top panel) and their respective specifications (bottom panel). In the rewarded phase, the median of all individual estimates is *r*_sb_ = .59, 95% CI [.41, .7], the range of estimates across specifications is [.14, .85], and 32.6 % of the estimates are above the minimum threshold of reliability (.7, following Nunnally, 1978). In the unrewarded phase, reliability estimates tended to be comparatively lower (median *r*_sb_ = .48, 95% CI [.3, .61], range [.0, .77], 6.9% above minimum threshold). Visual inspection of both phases shows that specifications that produce better estimates tend to include more blocks and use the mean instead of the median. In addition, it seems that there exists a pattern where filtering more outlier RTs using relative filters also leads to better estimates.

To further analyze how different specifications could affect reliability we performed a series of permutation tests^4^ (Holmes et al., 1996) considering their complex interactions and dependencies. Generally, the rewarded phase showed a higher reliability than the unrewarded phase (𝛥*r*_sb_ **=** .12, *p*_*perm*_ < .001), and the addition of more blocks progressively improves reliability (6 blocks - 2 blocks: 𝛥*r*_sb_ **=** .31, *p*_*perm*_ < .001; 12 blocks - 6 blocks: 𝛥*r*_sb_ **=** .17, *p*_*perm*_ < .001). Using the mean instead of the median significantly improved reliability across specifications (𝛥*r*_sb_ **=** .06, *p*_*perm*_ < .001), but log transformation of RTs did not result in a significant reliability improvement (𝛥*r*_sb_ **= –** . 01, *p*_*perm*_ = 0.265). When we looked at different methods for outlier removal, we found that removing RTs that were beyond 2 SDs resulted in improved reliability compared to when no relative filter was used (𝛥*r*_sb_ **=** .05, *p*_*perm*_ = .015), but the difference between using a 2 SDs or 3 SDs relative filter is not significant (𝛥*r*_sb_ **=** .02, *p*_*perm*_ = .136) nor the difference between 3 SDs and no relative filter (𝛥*r*_sb_ **=** .03, *p*_*perm*_= .128). To assess whether, in general, using a relative filter improved reliability compared to not using a relative filter, we collapsed the 2 SDs and 3 SDs filter specifications to compare those specifications regarding not using a relative filter, and showed that using a relative filter in general increased reliability across specifications (𝛥*r*_sb_ **=** .04, *p*_*perm*_ = .027). Other outlier removal methods did not affect reliability (removing two trials: 𝛥*r*_sb_ **=** .0, *p*_*perm*_ = .504; fixed filter: 𝛥*r*_sb_ **=** .0, *p*_*perm*_= .489). Finally, using a stricter cut-off of 70% accuracy significantly reduced reliability compared to use the 50% cut-off filter (𝛥*r*_sb_ **=** – . 04, *p*_*perm*_ = .018).

**Fig. 6:**
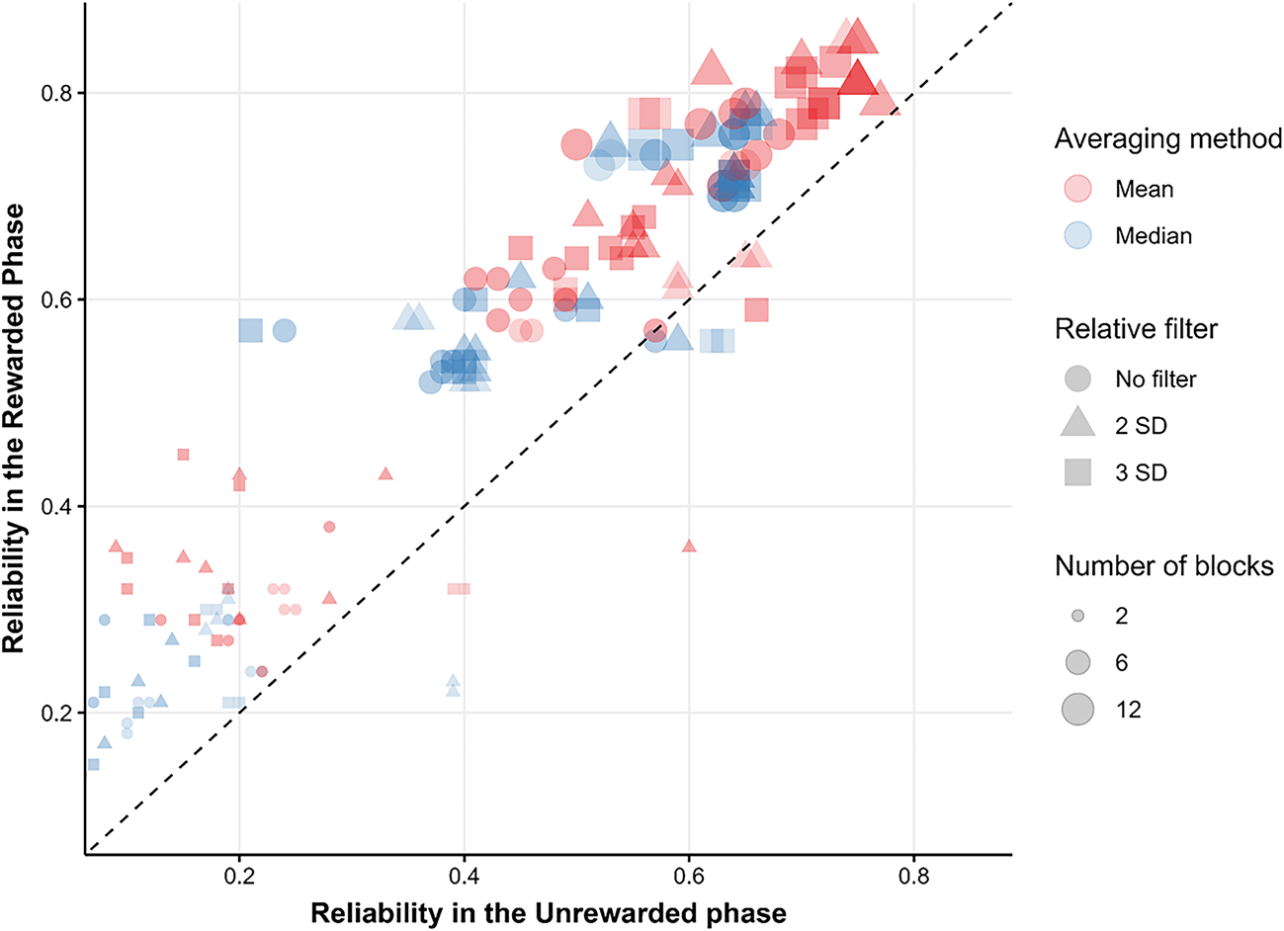
Comparisons of reliability between phases as a function of relevant pipelines. Split-half reliability for each pipeline in the rewarded phase plotted against the reliability achieved by the same pipeline in the unrewarded phase

The previous analyses confirm that reliability in the rewarded phase is in general superior to the unrewarded phase, and across phases reliability seems to be greatly influenced by the number of blocks used, the averaging method and the use of relative filters. To further visualize the impact of relevant pipelines over reliability, Fig. 6 shows how reliabilities in both phases differ as a function of number of blocks, the averaging method and the use of different relative filters. The figure shows that for almost every specification, the rewarded phase achieves better reliability than the unrewarded phase, and across phases, using the mean, computing the effect using 12 blocks and using 2 SDs as relative filter seems to give the best specifications. In fact, in both phases the specification with maximal reliability is mostly the same. When 12 blocks are used, the mean is employed as the averaging method, a 2SDs relative filter is used, no fixed filter is used, RTs are not log-transformed, the two first trials of each block are not removed, and the accuracy cut-off is .7 the reliability in the rewarded phase is *r*_sb_ = .85, 95% CI [.8, .88]. The maximal reliability in the unrewarded phase is achieved with the same set of specifications but when RTs are log-transformed (*r*_sb_ = .74, 95% CI [.67, .80]).

## Discussion

The first aim of the present study was to replicate and extend the results of Watson et al. (2019a), which demonstrated that the VMAC effect could be observed in conditions where a high reward predicting singleton had always been response irrelevant and held no informational value. In our study, we showed that the VMAC effect increased over time in the rewarded phase, that is, we observed a progressive increase in RTs when the high valued singleton acted as singleton distractor compared to the low valued singleton. Furthermore, when reward feedback was omitted in the unrewarded phase, the VMAC effect remained constant, without any evidence of reduction over trials. These two results replicate previous findings with Le Pelley’s paradigm (Le Pelley et al., 2015), where the VMAC effect emerged over trials, even when the reward-signals are always task irrelevant, and also replicate Watson et al. (2019a), who showed that once established, the VMAC effect remains present regardless of the current informational value of the reward-related stimuli. Beyond replicating Watson et al. (2019a), we extended the unrewarded phase to match the length of the rewarded phase. Explicitly informing participants about the uselessness of color to win points and increasing the length of the unrewarded phase allowed us to rule out a possible explanation that could drive the result of Watson et al. (2019a). If informational value about the color reward relationship is necessary to produce the VMAC effect, explicitly instructing participants that rewards will be discontinued should suffice to reduce or even abolish the VMAC effect. Here, as in Watson et al. (2019a), this was clearly not the case. However, in Watson et al. (2019a) it is possible that participants fail to pay sufficient attention to the instructions. In other words, it is possible that participants did not update their control attentional settings, leading to attentional biases for the high reward predictive stimulus. In our study, as the unrewarded phase had the same length as the rewarded phase, participants had sufficient direct experience to change their control attentional settings, even if they did not pay attention to the direct instructions. In contrast, we found no reduction of the VMAC effect over blocks in the unrewarded phase. These results suggest that once learned, the VMAC effect is resistant to Pavlovian extinction, even when the associated feature is always irrelevant and participants are explicitly informed about it. It is worth noting, however, that in our experiment, as in the original Watson et al. (2019a) study, participants were explicitly informed about the color reward contingency before the rewarded phase, which does not allow to rule out the possibility that the learning process behind the VMAC effect is in fact dependent on informational value. If this were the case, this result could perhaps be interpreted not in terms of Pavlovian learning, but as some sort of strategic form of attention (i.e., attending to color to gather information about the magnitude of the reward in the current trial) that could eventually become automatized with extensive practice (Theeuwes, 2018).

The second aim of this study was to explore the reliability of the VMAC effect measured in a rewarded learning stage (akin to Le Pelley's paradigm) and in an unrewarded test phase (akin to Anderson's paradigm), and to explore how different specifications can impact reliability estimates. Our analysis shows that across 288 different specifications, there is substantial heterogeneity in reliability. Comparing both phases, it seems that for almost every specification, the rewarded stage shows higher reliability than the unrewarded phase (𝛥*r*_sb_ **=** .l2). Furthermore, for both phases, as expected following classical test theory, reliability improved with the number of blocks, showing that the higher estimates of reliability are always reached when all blocks are included. Given that the VMAC effect increases through blocks in the rewarded phase, this result also means that specifications that maximize the effect size (i.e., calculating the effect in the two last blocks) produce the worst reliability estimates, at least during the learning phase. This negative relationship between the effect size and reliability is not surprising; in fact, it is in accordance with the so-called reliability paradox (Hedges et al., 2018), by which tasks (or specifications) that produce more robust effects at the group level often show poor reliability. Although it makes sense to focus on later trials to confirm that learning has taken place, our multiverse shows that if the goal of the study is to detect individual differences, then task length should be planned to enhance reliability. That is, experiments that seek to use VMAC as a measure of individual differences should take into account that the procedures that maximize the size of the effect are not necessarily the same ones that best capture individual differences. For the same reasons, although removing participants behaving at chance might increase the size of the VMAC effect, including them might improve reliability and therefore facilitate the detection of individual differences across participants. Nevertheless, it is worth noting that participants who behave near chance may not be paying sufficient attention to the task, and thus variations in the observed VMAC effect might not reflect true variation in susceptibility to VMAC. For example, it is possible that including these participants in the analysis artificially increases internal consistency by adding a few extreme data points in the calculation of split-half reliability. As we only excluded 13 participants with less than 70% accuracy, a post hoc analysis with this subset of participants is not advisable, but we raise the possibility that including participants who behave close to chance may not necessarily result in a more valid measure, even if it yields higher reliability estimates.

Although the present study confirms that reliability increases as more blocks are included in the analysis, it does not take into account other relevant questions related to the quality of the trials that are included or excluded from the analysis. It could be the case that the removal of trials where learning is not yet stabilized leads to better reliability estimates. Our analyses show that, at the group level, the VMAC effect is not significant in the first two blocks of trials. In implicit learning paradigms, this could be an important factor, because if learning is not yet stabilized in early stages, it is possible that those first trials map onto a different latent construct than the rest of the task, thus increasing noise and reducing reliability. To rule out that possibility, we re-ran the multiverse analysis for all the specifications where the 12 blocks of trials are included, but we varied whether the first two blocks of the rewarded stage were included or not. The reliability estimates of this specification are shown in Fig. S5 of the Supplementary Material. We ran a permutation test to compare if removing these first two blocks would increase reliability compared to using all valid trials. The results showed that removing those trials produces a small but significant reduction in reliability (𝛥r_sb_ **= –** .02, *p*_*perm*_ < .01). This result is in accordance with a recent reliability multiverse analysis in another implicit learning effect, contextual cueing of visual attention, where reliability estimates always improved with the inclusion of any epoch of the task, independently of whether those epochs correspond to early or later stages of the task (Vadillo et al., 2023). Taken collectively, these results suggest that it is not advisable to exclude any subset of trials from the calculation of the measure.

**Fig. 7:**
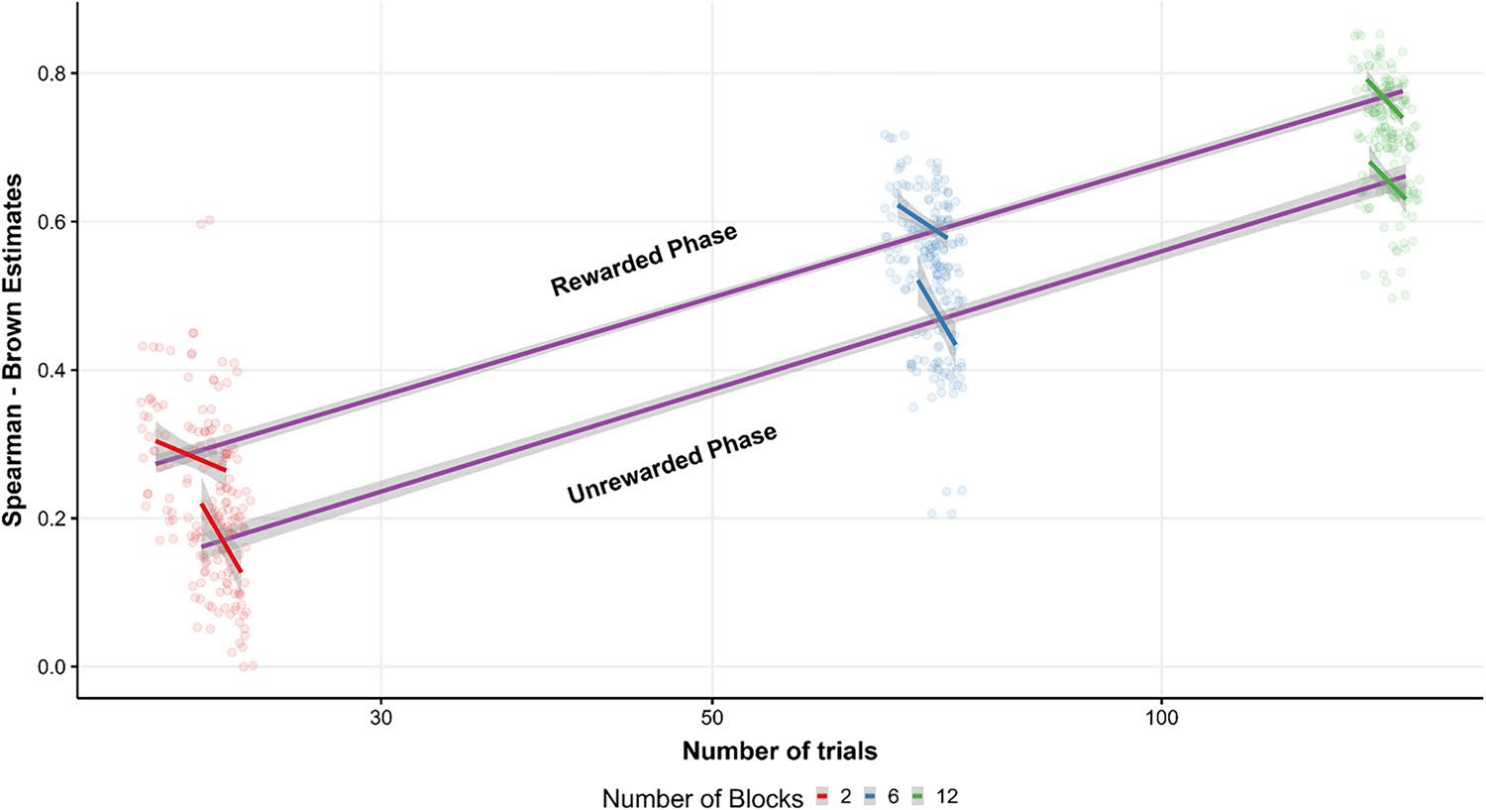
Relationship between reliability and number of trials. The general relationship between the overall number of trials (log-scaled for visualization purposes) and reliability is represented by the *blue regression line* (95% CI), where the *upper regression line* denotes the rewarded phase and the *lower line* denotes the unrewarded phase. *Color* represents a different number of blocks used for reliability calculation, where each colored regression line shows how outlier removal methods influence on reliability across different numbers of blocks

Interestingly, although reliability generally improves with the number of observations, Parsons (2022) showed that specifications with more stringent trial selection criteria tend to produce better reliability estimates. We have seen a similar pattern in our multiverse analysis, where the most stringent relative filter (i.e., filtering RTs by 2 SDs) produces better estimates. To visualize this idea, in Fig. 7 we show the overall relationship between number of trials and reliability. Although there is a general positive relationship between the number of trials and reliability, if we look separately to each level of number of blocks, there is actually a negative relationship between the number of trials and reliability, with fewer trials leading to better estimates. The fact that other methods of outlier removal, such as using a fixed filter or removing the first two trials of each block, did not have a large influence on reliability suggests that in specifications where a relative filter is employed, the use of an additional trial selection criteria could be redundant. These results suggest that it is not only important to optimize the task length, but it is also important to choose a good method of outlier removal. Furthermore, the present study together with other multiverse reliability analyses on different experimental tasks (Parsons, 2022; Vadillo et al., 2023) seems to support the conclusion that the optimal decision for outlier removal may vary between experimental and correlational research. For instance, if the emphasis of a particular study is on group-level effects, it is advisable, in accordance with Miller (2023), to avoid outlier removal procedures, as these could introduce bias and diminish statistical power. Conversely, in individual differences studies, employing a robust outlier removal procedure may enhance reliability and possibly attenuate the impact of measurement error.

**Fig. 8:**
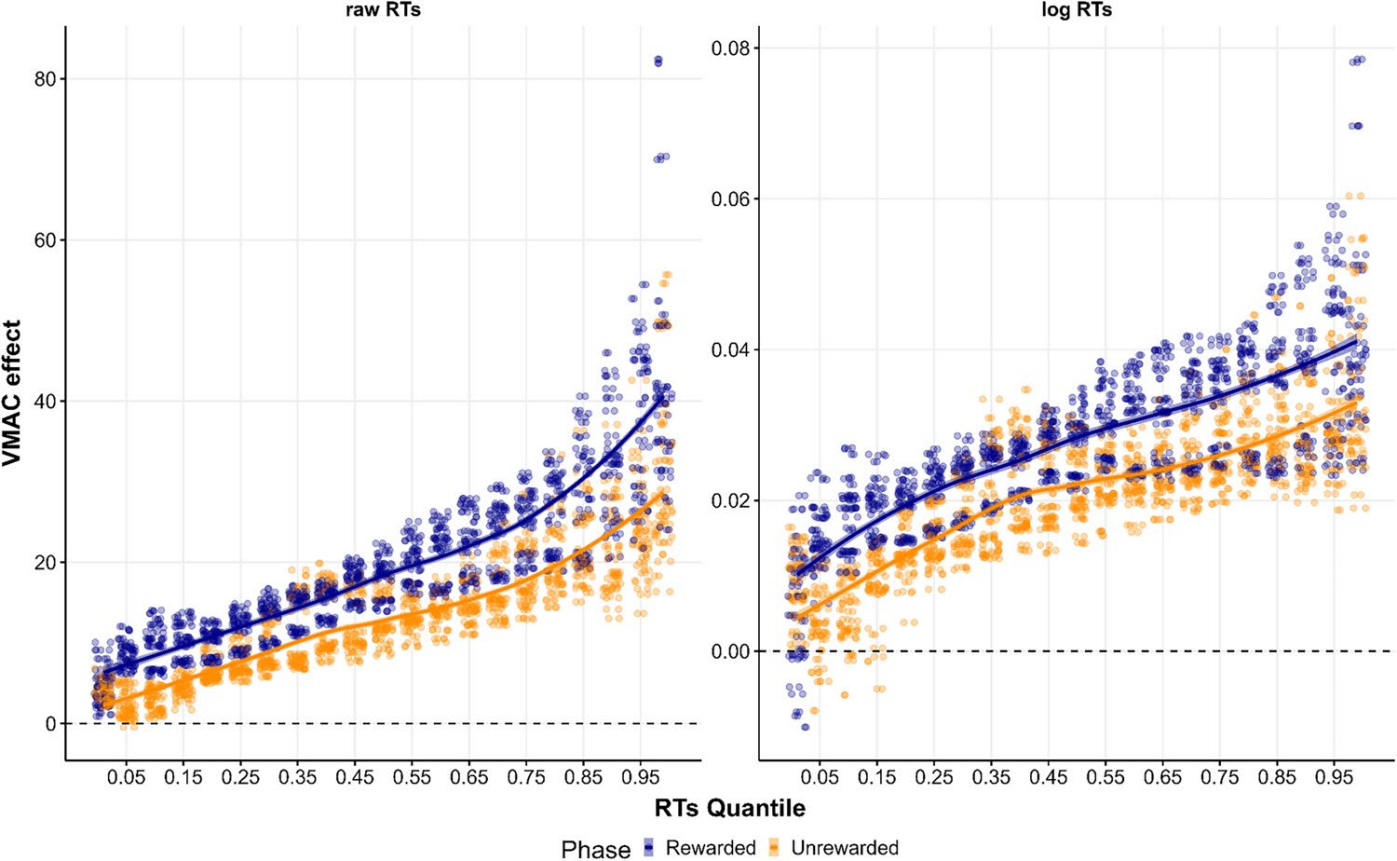
VMAC effect calculated in different quantiles of the RTs distribution. Each *point* represents the bootstrapped mean VMAC effect at different quantiles of the RTs distribution (10,000 replications) in the 144 datasets generated for the multiverse analysis. On the right are depicted the data sets where RTs were log-transformed, while on the left are the data sets where the RTs are raw. The *regression line* shows LOESS fit, which represents the relationship between the effect and the quantile used to calculate the effect. *Color* represents the phase used to calculate the effect

Lastly, it seems that the averaging method makes a substantial difference in reliability. One plausible explanation for this result is that, given that the mean is more sensitive to the tails of the RTs distribution, it is possible that the VMAC effect is partly driven by an increase of RTs in the tail of the distribution when a high-reward singleton appears in the display. To explore this possibility, we computed the size of the VMAC effect using different quantiles of the RTs distribution to aggregate the data, instead of using the mean or the median. We applied this strategy to all the datasets generated for the multiverse analyses. The results are shown in Fig. 8. As can be seen, there is a general increase in the size of the VMAC effect on slower responses. This suggests that our guess could be correct. To further support this claim, we decided to run another multiverse analysis to test whether using a higher quantile could improve reliability compared to using the median. Arbitrarily, we decide to use the .75 quantile. The reliability estimates of this new specification are shown as Supplementary Material (Fig. S6). A permutation test shows that using a higher quantile does not improve reliability compared to using the median (*p*_*perm*_ = .490). Although Fig. 8 shows that the general effect size is bigger at higher quantiles, there may also be more variability in the tails of the distribution, which produces random variation in the VMAC effect which ultimately hampers reliability. Thus, perhaps the reason why the mean is more reliable than the median is simply that it is more sensitive to the general shape of the RT distribution, not just the tails. Given the present finding, future research should address systematically why using the mean is more beneficial for reliability than using the median.

As explained in the introduction, previous studies of the psychometric properties of the VMAC effect do not support the use of RTs measures to study individual differences due to their apparent lack of stability (Anderson & Kim, 2019; Friedrich et al., 2023), but this does not necessarily mean that their internal consistency is too low for correlational research (see Ivanov et al., 2023). Our multiverse analysis showed that, depending on preprocessing pipelines, the internal consistency of both phases can vary enormously. Although in most cases reliability was below the typical threshold of reliability for individual differences studies, under some specifications reliability could be considered acceptable. In fact, in the rewarded phase of the present study, if all the trials available are included in the calculations, reliability is systematically above .7. Sadly, several studies measuring individual differences in VMAC have restricted their analyses to a small subset of the available data (Albertella et al., 2019, 2020a, 2020b; Liu et al., 2021), an analytic decision that could yield reliability levels as low as .14 in the rewarded phase and almost 0 in the unrewarded phase. This raises concerns about the potential impact of measurement error in previous studies that have used a similar preprocessing pipeline. Based on the present findings, as some preprocessing pipelines can produce measures with relatively high internal consistency, future research aimed at investigating individual differences in the VMAC effect should include data preprocessing pipelines that are expected to produce higher internal consistency and, if possible, explore the impact of equally valid preprocessing pipelines on potential inferences. As suggested by Parsons (2022), a multiverse reliability analysis could also be performed as a robustness check.

Although previous studies have raised reliability concerns about the use of experimental tasks in correlational research (Draheim et al., 2019; Hedges et al., 2018; Rouder & Haaf, 2019), the present study shows that with the appropriate preprocessing pipelines, the measures taken in experimental tasks can reach acceptable reliability levels. However, it is important to note that our reliability estimates may not generalize to other assessments of the same measure. Reliability is not a property of the instrument, it is a property of the measure, and reporting reliability of experimental measures is not a standard in psychological science (Parsons et al., 2019). For that reason, we want to raise awareness about the necessity of reporting the reliability of the VMAC task when the objective of the study is to explore individual differences, so that the results can be informative, transparent, and replicable. Fortunately, reporting reliability in experimental tasks has become a trivial matter due to the effort of the scientific community to develop software that facilitates the estimation of reliability through different methods (Parsons, 2021; Pronk et al., 2022). Once reporting reliability becomes a standard practice, a promising next step would be to study the expected reliability across a relatively large set of published studies by means of a reliability meta-analysis, as has been done in other paradigms, such as the implicit association test (Hussey & Drake, 2020).

While reporting reliability and adopting preprocessing pipelines that optimize reliability are valuable practices, these steps alone may not be sufficient to mitigate the impact of measurement error on studies of individual difference. It would also be beneficial to utilize alternative strategies such as incorporating specific task design features that enhance reliability (Rey-Mermet et al., 2019; Siegelman et al., 2017) or implementing analytical methods that take measurement error into account (Haines et al., 2020; Malejka et al. 2021; Rouder & Haaf, 2019). Future research could investigate the influence of distinct design features on task reliability. Studies of VMAC vary enormously in different aspects of the task whose impact on reliability is so far unknown. For instance, in Anderson's original paradigm the learning phase of the reward schedule is probabilistic (i.e., when the high color distractor appears on-screen, there is a certain probability that reward will be higher), while in Le Pelley’s paradigm the reward schedule is deterministic (i.e.,. the number of points earned is always larger when the reward high singleton is presented). The learning process can occur under instrumental (i.e., participants have to select a certain stimulus to earn reward) or Pavlovian conditions (i.e., stimuli merely signal the magnitude of the reward but do not require a direct response). In some variants of the task, only gains are possible, while in others both gains and losses can occur. Experiments can also include or exclude absent trials from the search task. These and other design decisions could significantly influence reliability.

In summary, in this study we have replicated and extended the study by Watson et al. (2019a) with an online version of the task, showing that the VMAC effect is robust to the omission of contingencies during the testing stage. Furthermore, we have explored how different preprocessing decisions can affect reliability estimates in a study with a design analogous to the two most common paradigms used to measure the effect. The results show high heterogeneity, highlighting the need to design individual difference studies on the basis of data preprocessing decisions that maximize reliability. We recommend that researchers working with this effect take the standard practice of reporting the reliability of their measures. However, on its own, this does not address all the possible implications of measurement error and efforts should be made in future studies to find alternatives that either improve reliability or take measurement error into account.

### Supplementary information

ESM 1 (DOCX 2290 kb)

## Footnotes

1 Note that in Awh et al. (2012) the authors refer to top-down and bottom-up processing. Some authors such as Gaspelin and Luck (2018) use the term top-down to refer to any situation where context, learning or expectations modulate perception and attention. By such a definition, selection history could be defined as “top-down”. For the sake of clarity, here we used the terms goal-directed and stimulus-driven instead of the classical terminology employed by Awh et al. (2012).

2 The model formula uses the notation implemented by the lme4 package (Bates et al., 2015), where the parentheses denote the random effect structure and the rest of the right-hand side formula indicates the fixed effects.

3 In the main text and in every figure in the manuscript, we present model predictions and conditional effect back-transforming log (RTs) to the response scale. Model predictions for singleton conditions are computed averaging predictions across all blocks.

4 The permutation test is a non-parametric method that involves comparing two conditions (i.e., specifications that use the mean vs. specifications that use the median) against a null hypothesis distribution, which is generated by randomly assigning the labels of the two conditions to various specifications and calculating the ensuing difference. The null distribution is obtained by randomly shuffling the labels of a given condition 10,000 times. Afterward, the observed difference is compared with this null distribution. The observed difference is deemed significant if it falls in the most extreme 5% of the null distribution (i.e., if it is more extreme than 95% of the values in the null distribution). In the main text, *p* values for a permutation test are referred to as *p*_*perm*_.
