# Supplementary material for: On the reliability of value-modulated attentional capture: An online replication and multiverse analysis
Source: Behav Res Methods. 2024 Jan 9;56(6):5986–6003. doi: 10.3758/s13428-023-02329-5 (PMC11335866; doi:10.3758/s13428-023-02329-5)
Supplement: Supplementary file 1 — (DOCX 2290 kb) [file 13428_2023_2329_MOESM1_ESM.docx]

# Supplementary Material

## Reliability multiverse analysis for the attentional capture effect

For the interested reader, we present here a similar multiverse analysis of split-half reliability for the attentional capture (AC) effect, defined as the difference in RT for the low singleton trials minus the absent singleton trials. Since the temporal pattern of the effect is different from that of the VMAC effect (Figure 3 in the main text), we decide to change which trials were selected in the rewarded phase compared to the multiverse presented in the main text. Specifically, since the AC effect in the rewarded phase seems to decrease with time, selecting the earliest trials could potentially increase reliability, as participants may be more susceptible to presenting AC for the singleton distractor at the beginning of the task. Therefore, for both phases, we varied whether the first two blocks, the first six blocks, or all 12 blocks were subset. All other factors remained unchanged with respect to the analysis presented in the main text, resulting in the same 288 specifications.

Figures S1 and S2 show the curve of reliabilities for the AC effect in the rewarded and unrewarded phases, respectively. The reliabilities are sorted in ascending order (top panel) and their respective specifications (bottom panel) in both figures S1 and S2. For the rewarded phase, the median reliability from the specification curve is *r*_sb_ = .47 95% CI[.3, .59], the range of specifications is [.07, .82], and 7.99% of them are above the recommended reliability threshold. In contrast, in the unrewarded phase, the specification curve shows that reliability tends to be lower (median *r*_sb_ = .29; 95% CI[.07, .46]; range: [.06, .56]; 0% above minimum threshold).

**Figure S1**

*Spearman – Brown estimates across the rewarded phase for the AC effect*

*
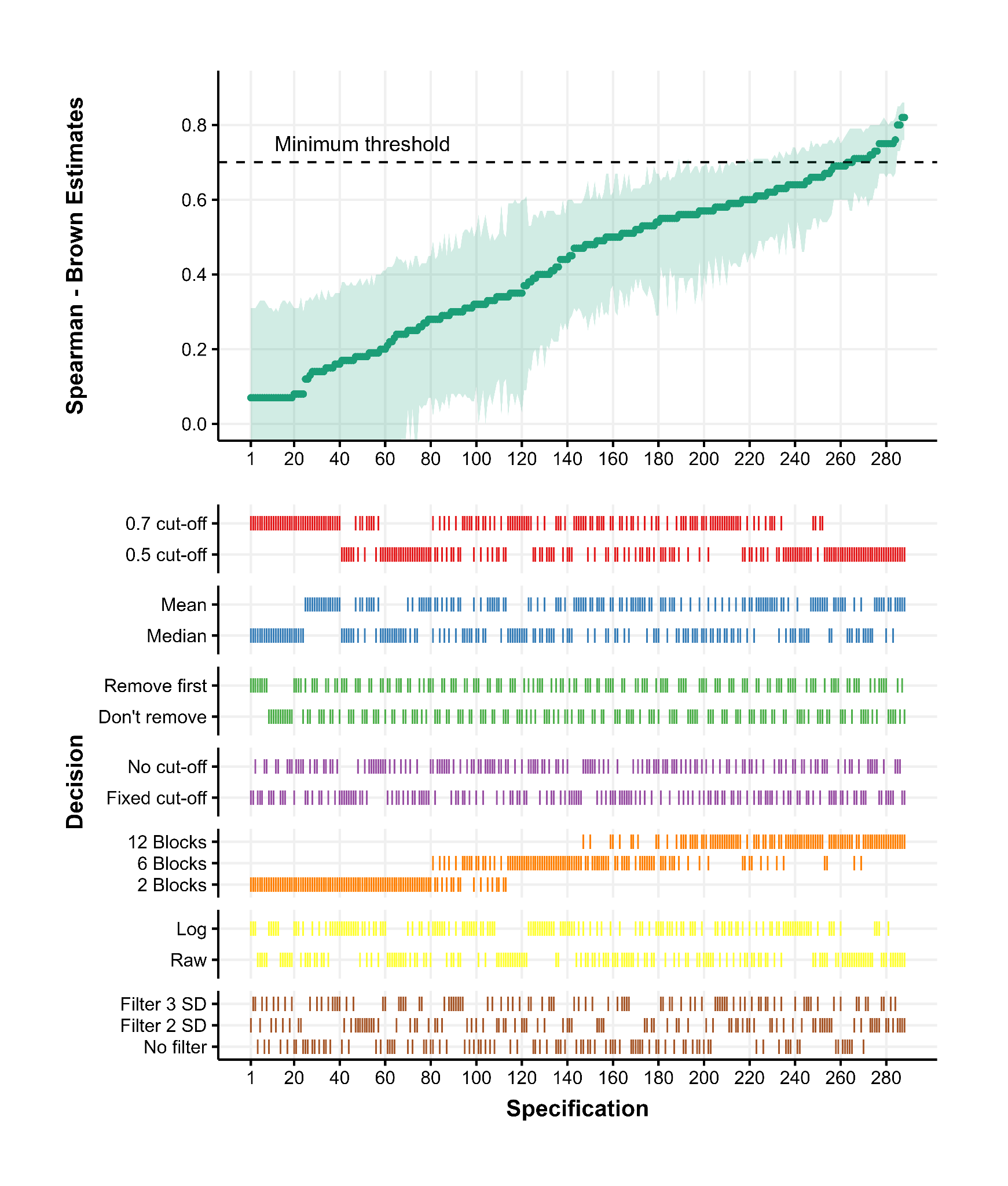
*

As in the main text, to explore the impact of different preprocessing decisions on the AC effect, we also used a series of permutation tests to analyze all sets of specifications. First, as can be seen in Figures S1 and S2, the reliability of the AC effect is higher in the rewarded phase than in the unrewarded phase (𝛥*r*_sb_ **=** .13, *p_perm_* < .001). As in the previous multiverse, including more blocks in the analysis leads to higher reliabilities (6 blocks - 2 blocks: 𝛥*r*_sb_ **=** .17, *p_perm_* < .001; 12 blocks - 6 blocks: 𝛥*r*_sb_ **=** .11, *p_perm_* < .001). However, as suggested by the specification curve in Figure S1 and Figure S2, this increase in reliability is more accentuated in the rewarded phase (6 blocks - 2 blocks: 𝛥*r*_sb_ **=** .17, *p_perm_* < .001; 12 blocks - 6 blocks: 𝛥*r*_sb_ **=** .11, *p_perm_* < .001) compared to the unrewarded phase (6 blocks - 2 blocks: 𝛥*r*_sb_ **=** .07 *p_perm_* < .001; 12 blocks - 6 blocks: 𝛥*r*_sb_ **=** .05, *p_perm_* < .01). Again, it seems that using the mean rather than the median as the averaging method seems to produce higher reliabilities (𝛥*r*_sb_ **=** .09, *p_perm_* < .001), but using raw compared to log-transformed RTs does not seem to significantly affect reliability (𝛥*r*_sb_ **=** .02, *p_perm_* = .059). While the use of fixed filters does not significantly impact reliability estimates (Fixed RT filter: 𝛥*r*_sb_ **=** .008, *p_perm_* = .30; Remover first two trials: 𝛥*r*_sb_ **=** .0001, *p_perm_* = .5), the use of a relative filter with 2SDs (𝛥*r*_sb_ **=** .04, *p_perm_* < .01) or 3SDs (𝛥*r*_sb_ **=** .05, *p_perm_* < .01) produced a significant increase in reliability, but there were no differences between a 2SDs or 3SDs filter (𝛥*r*_sb_ **= -**.005, *p_perm_* = .39). Finally, as in the multiverse for the VMAC effect, using a more stringent accuracy filter of 70% negatively affects reliability (𝛥*r*_sb_ **= -**.12, *p_perm_* < .001).

**Figure S2**

*Spearman – Brown estimates across the unrewarded phase for the AC effect*

*
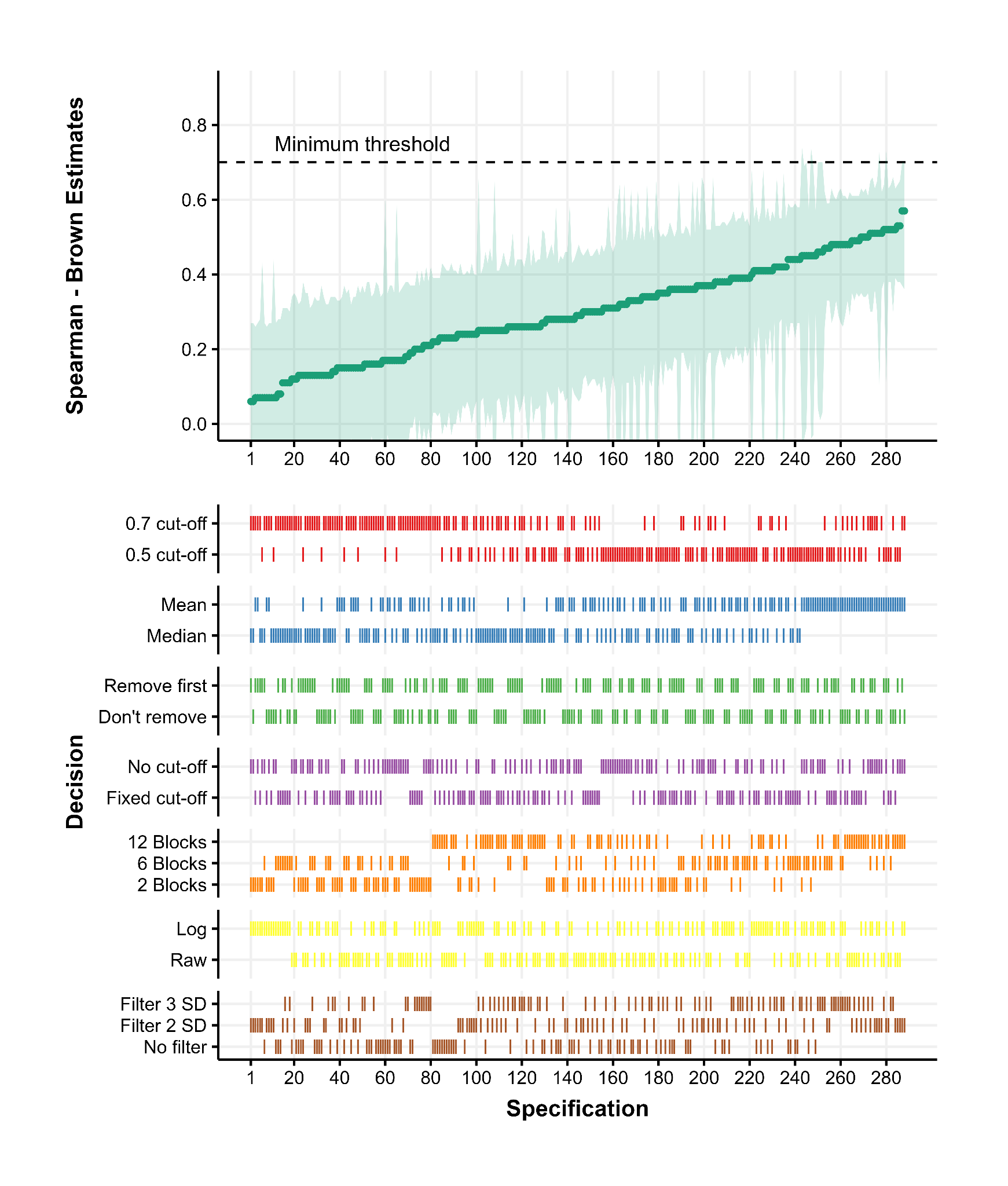
*

The previous analysis seems to converge with the conclusions presented in the main text. In order to visualize how different preprocessing decisions affected the AC effect in both phases of the task, Figure 3 plots reliabilities in the rewarded phase against the reliabilities of the unrewarded phases, as Figure 6 in the main manuscript. The specification with maximum reliability for the rewarded phase is achieved when 12 blocks are used, the mean is used as the averaging method, a 2SDs relative filter is used, a fixed filter is used, RTs are not log-transformed, the first two trials of each block are not removed, and the accuracy cut-off is .5 (*r*_sb_ = .82 95% CI[.76, .86]). The maximum reliability specifications for the unrewarded phase are largely the same, but RTs are log-transformed and an accuracy cut-off of .7 is used instead (*r*_sb_ = .56 95% CI[.76, .86]).

**Figure S3**

*Comparisons of Reliability between phases as a function of relevant pipelines in the AC effect*


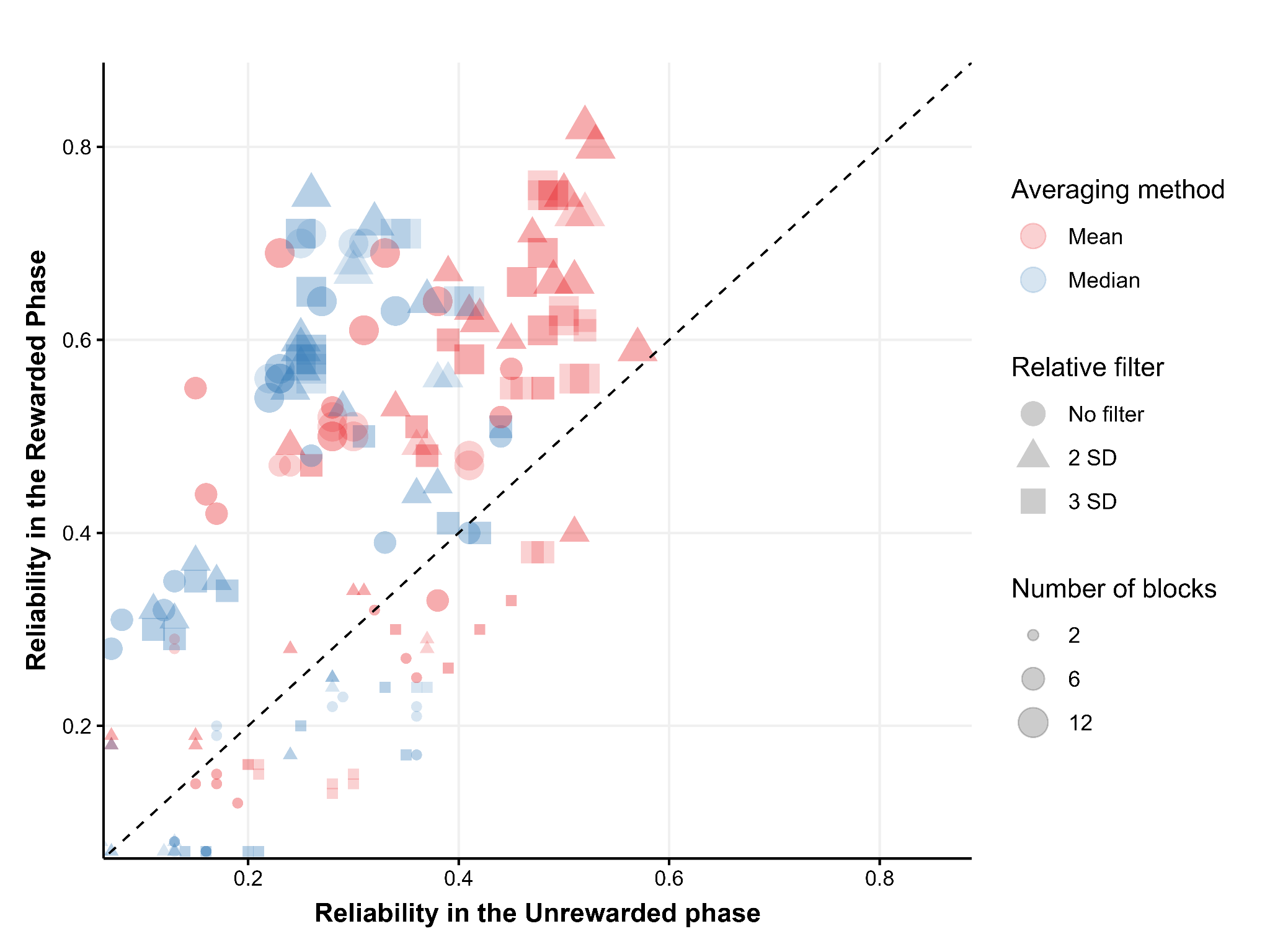


Since we found a similar effect in the use of relative filters over RTs as in the VMAC effect multiverse, to visualize the same idea as in Figure 7 of the main manuscript, in Figure S4 we have plotted the general relationship between the number of blocks included and reliability, and within each level of the block factor, how removing more trials affects reliability. As can be seen, the general pattern is very similar to that of the VMAC effect, showing that in general more trials lead to better reliability, but within each level of the block factor, removing outliers seems to increase reliability. On the other hand, the slopes of the linear fit between the two phases of the task reflect that using more blocks to compute the effect in the unrewarded phase leads to a reduced increase in reliability compared to the rewarded phase.

**Figura S4**

*Relationship between reliability and number of trials in the AC effect*


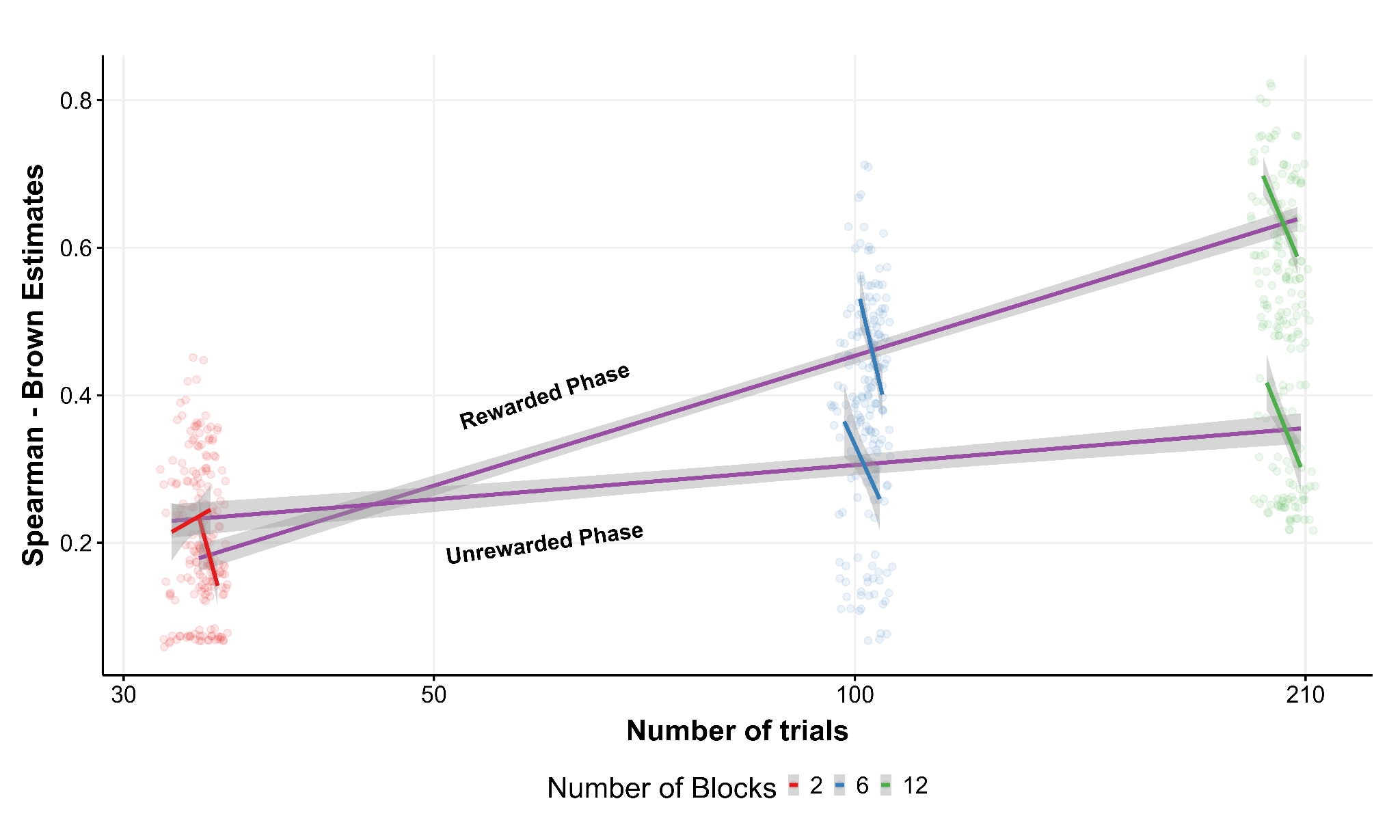


Finally, it is tempting to interpret these results as a direct comparison of the internal consistency of the VMAC effect with the AC effect. However, the problem with this comparison is that the present design is unbalanced in terms of high and low singleton trials compared to absent trials. There are 240 singleton trials (half high and half low singleton) in each phase, whereas there are only 48 absent trials, which means that the absent condition is expected to be measured with less precision. Although a direct comparison is not advisable, it can be seen that the reliability of both the VMAC and AC effects are similarly affected by the same factors, which may suggest that these results could be extrapolated to measures of attentional capture more generally.

## Complementary multiverse analysis for the VMAC effect

**Figure S5**

Multiverse to compare the effect of removing the first two blocks over reliability

**
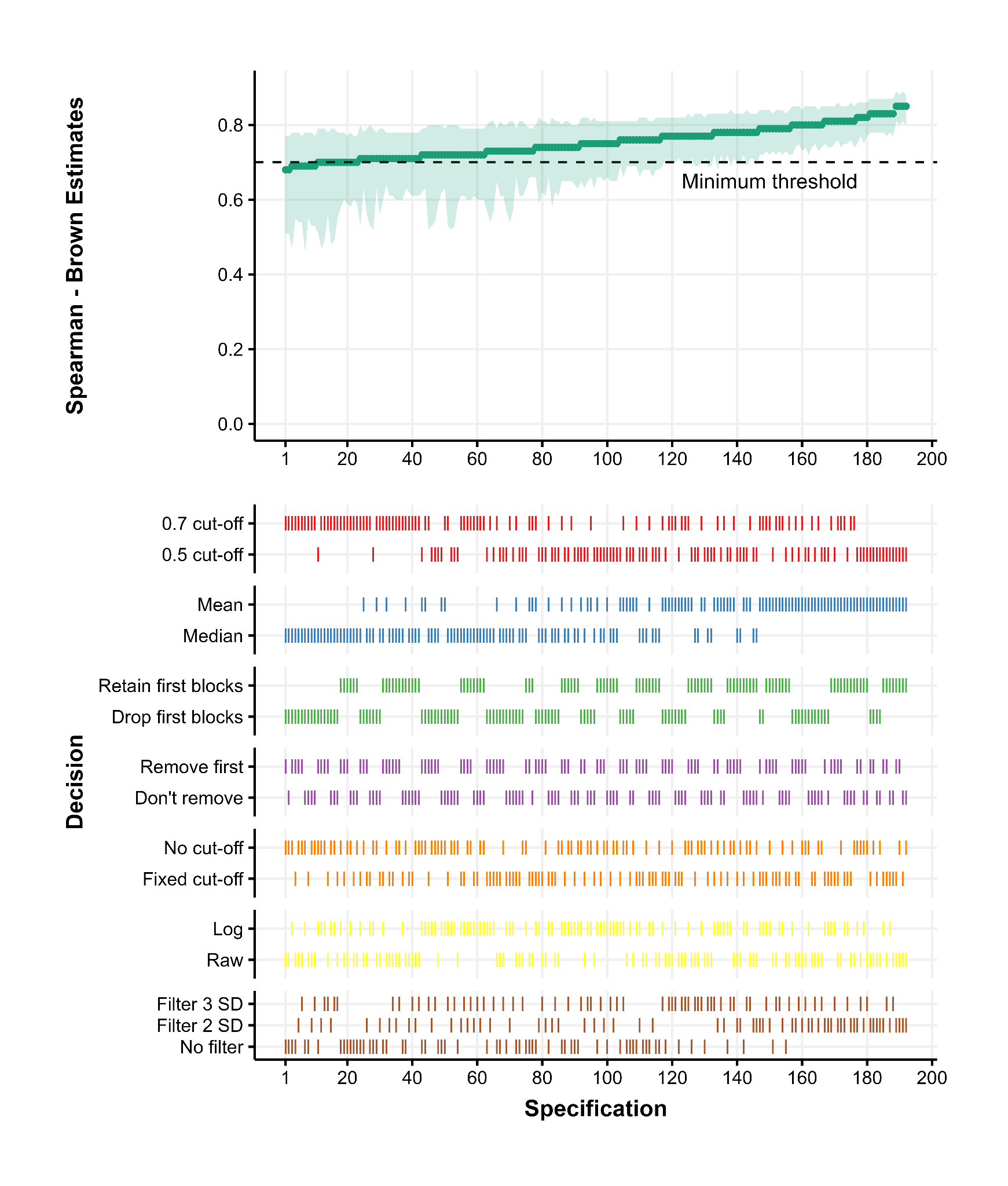
**

**Figure S6**

Multiverse to compare median vs. .75 quantile averaging method

**
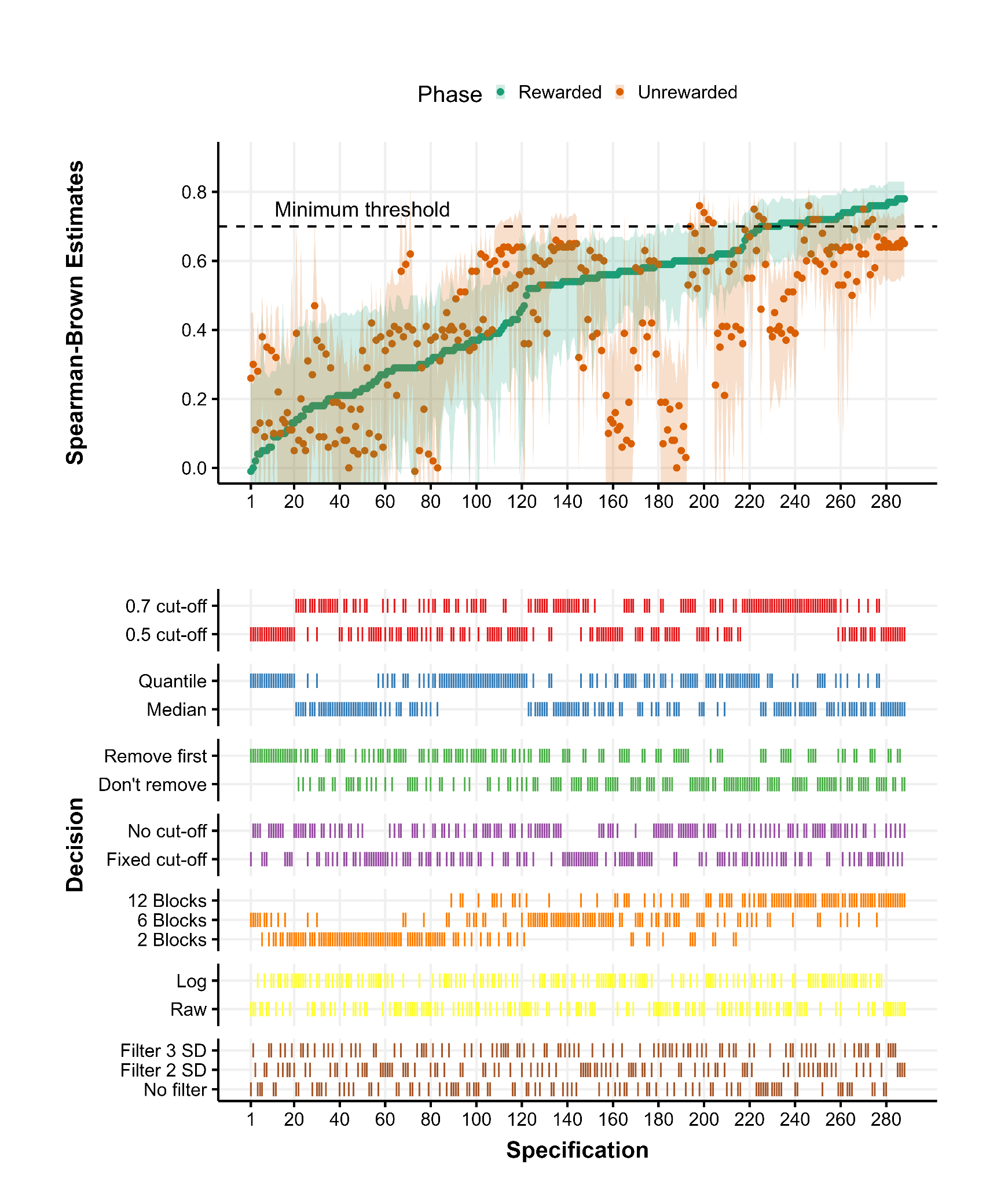
**

*Note.* The reliabilities for both phases are sorted in ascending order by the reliabilities of the specifications in the rewarded phase (top panel) and their corresponding specifications (bottom panel).
